# Serum metabolites as biomarkers in systemic sclerosis-associated interstitial lung disease

**DOI:** 10.1038/s41598-020-78951-6

**Published:** 2020-12-14

**Authors:** C. Meier, K. Freiburghaus, C. Bovet, J. Schniering, Y. Allanore, O. Distler, C. Nakas, B. Maurer

**Affiliations:** 1 Department of Rheumatology, Center of Experimental Rheumatology, University Hospital Zurich, Zurich, Switzerland; 2 University Institute of Clinical Chemistry, Bern University Hospital, University of Bern, Bern, Switzerland; 3 Department of Rheumatology A, Descartes University, APHP, Cochin Hospital, Paris, France; 4 Laboratory of Biometry, University of Thessaly, Volos, Greece

**Keywords:** Biomarkers, Molecular medicine, Systemic sclerosis, Translational research

## Abstract

Systemic sclerosis (SSc) is a severe multi-organ disease with interstitial lung disease (ILD) being the major cause of death. While targeted therapies are emerging, biomarkers for sub-stratifying patients based on individual profiles are lacking. Herein, we investigated how levels of serum metabolites correlated with different stages of SSc and SSc-ILD. Serum samples of patients with SSc without ILD, stable and progressive SSc-ILD as well as of healthy controls (HC) were analysed using liquid targeted tandem mass spectrometry. The best discriminating profile consisted of 4 amino acids (AA) and 3 purine metabolites. l-tyrosine, l-tryptophan, and 1-methyl-adenosine distinguished HC from SSc patients. l-leucine, l-isoleucine, xanthosine, and adenosine monophosphate differentiated between progressing and stable SSc-ILD. In SSc-ILD, both, l-leucine and xanthosine negatively correlated with changes in FVC% predicted. Additionally, xanthosine was negatively correlated with changes in DLco% predicted and positively with the prognostic GAP index. Validation of l-leucine and l-isoleucine by an enzymatic assay confirmed both the sub-stratification of SSc-ILD patients and correlation with lung function and prognosis score. Serum metabolites may have potential as biomarkers for discriminating SSc patients based on the presence and severity of ILD. Confirmation in larger cohorts will be needed to appreciate their value for routine clinical care.

## Introduction

Systemic sclerosis (SSc) is a multi-system fibrotic disease with high morbidity and mortality^1^. Fibrosing interstitial lung disease (ILD) affects 60% of patients and is the leading cause of disease-related deaths^2,3^. Disease severity at time point of ILD diagnosis and progression over time determine the prognosis^2,4,5^. Currently available measures such as imaging, pulmonary function tests, or patient reported outcome scores have been shown to be of limited predictive value for the identification of SSc-ILD patients with poor prognosis^6,7^. Circulating biomarkers as readily available tools have been studied extensively with focus on serum proteins and auto-antibodies, but have not yet been established for daily use^8^.

A more recent research trend focuses on metabolic changes in diseased states^9^. Metabolic pathways represent central hubs within cells that have significant impact on complex signalling networks as well as cell functions. Metabolites are the final products of pathophysiologic processes caused by diseased states or by exposure to environmental factors or drugs and as such offer an integrated view of the biochemical milieu of bodily fluids or tissues^10^. The profiling of the metabolites present in biological samples—called *metabolomics—*has the potential to provide novel insights into pathophysiological processes^11^. Disturbed metabolism in the setting of (lung) fibrosis is increasingly acknowledged^12^. In general, the balance shifts from a catabolic to an anabolic state. In accordance, corresponding changes in the respective energy pathways occur, including e.g. an altered amino acid (AA) metabolism and enhanced protein production, an increased glycolysis with depletion of glucose, oxidative stress with mitochondrial dysfunction, decreased ATP levels, and reduced beta-oxidation of fatty acids^13–15^. The biologic relevance is supported by recent data showing that metabolic reprogramming successfully retransforms myofibroblasts to fibroblasts and activates degradation of extracellular matrix^16–18^, which offers novel therapeutic perspectives.

Metabolomics studies in ILD have so far mainly analysed lung tissue, bronchoalveolar lavage fluid (BALF) or exhaled breath condensate (EBC) from patients with idiopathic pulmonary fibrosis (IPF), the most prevalent form of ILD^14,19^. One serum-based study identified LysoPC, a potential precursor of lysophosphatic acid as discriminator between patients and healthy controls^20^. In SSc, data are scarce. Two recent studies showed some diagnostic potential since SSc patients could be distinguished from healthy controls based on their serum and/or urine metabolome with alterations in hexose, glycerolipid, and amino acid metabolism^21,22^.

Given the high unmet need of sub-stratifying biomarkers for ILD in SSc, the aim of our study was to evaluate the potential of serum metabolites to identify SSc patients according to their disease state with special focus on differentiating those with progressive from non-progressive ILD as base of therapeutic decision making.

## Results

### Patients’ characteristics

To decrease potential confounding factors for metabolomics studies, we matched the participating individuals (n = 12 per group, n = 48 in total) for age, sex, and time point of blood withdrawal. Demographic, clinical, and laboratory data are summarized in Table 1. The mean age was 61.5 ± 9.9 years, the majority of patients was female (n = 30, 83.3%), 25.0% (n = 9) had diffuse cutaneous and 27.8% (n = 10) had limited cutaneous disease, whereas 22.2% (n = 8) and 25.0% (n = 9) had only sclerodactyly or no skin involvement, respectively. The mean disease duration of SSc defined as onset of first non-Raynaud’s phenomenon was 9.0 ± 7.7 years. The mean disease duration of ILD was 3.2 ± 2.5 years. No differences in non-lung organ involvement were observed.

**Table 1:** Clinical characteristics of SSc patients of the Zurich cohort at time point of serum collection.

Characteristics	Non-ILD SSc (n = 12)	Stable SSc-ILD (n = 12)	Progressive SSc-ILD (n = 12)	All (n = 24–36^a^)
**General**				
**Sex, n (%)**				
Female	10 (83.3)	10 (83.3)	10 (83.3)	30 (83.3)
Male	2 (16.7)	2 (16.7)	2 (16.7)	6 (16.7)
Age (mean ± SD)	61.6 ± 9.1	62.0 ± 10.3	63.1 ± 11.1	61.5 ± 9.9
BMI (mean ± SD)	25.7 ± 5.8	24.2 ± 4.5	22.7 ± 4.3	24.2 ± 4.9
**SSc**				
SSc disease duration^b^, years (mean ± SD)	10.0 ± 10.0	7.1 ± 5.5	7.0 ± 9.9	9.0 ± 7.7
mRSS (mean ± SD)	3.7 ± 4.1	4.5 ± 5.8	10.3 ± 7.7	6.2 ± 6.6
**Extent of skin disease, n (%)**				
dcSSc	1 (8.3)	2 (16.7)	6 (50.0)	9 (25.0)
lcSSc	4 (33.3)	2 (16.7)	4 (33.3)	10 (27.8)
Sclerodactyly only	2 (16.7)	4 (33.3)	2 (16.7)	8 (22.2)
No skin involvement	5 (41.7)	4 (33.3)	0 (0)	9 (25.0)
**Joint involvement, n (%)**				
Synovitis	3 (25.0)	0 (0)	3 (25.0)	6 (16.7)
Tendon friction rubs	2 (16.7)	0 (0)	0 (0)	2 (0.06)
**Organ involvement, n (%)**				
Cardiac disease^c^	1 (8.3)	1 (8.3)	1 (8.3)	3 (8.3)
History of renal crisis	0 (0)	0 (0)	1 (8.3)	1 (0.03)
Gastrointestinal involvement^c^	7 (58.3)	4 (33.3)	7 (58.3)	18 (50.0)
**Autoantibody positivity, n (%)**				
Anti-centromere	9 (75)	2 (16.7)	1 (8.3)	12 (33.3)
Anti-topoisomerase 1	1 (8.3)	2 (16.7)	7 (58.3)	10 (27.8)
**Markers of systemic inflammation (mean ± SD)**				
ESR, mm/h	11.8 ± 8.4	25.3 ± 20.1	26.5 ± 20.6	20.9 ± 17.9
CRP, mg/L	3.1 ± 2.6	2.6 ± 3.5	4.4 ± 4.9	3.3 ± 3.7
Disease activity index (mean ± SD)	2.56 ± 0.92	2.41 ± 2.18	3.59 ± 2.18	2.91 ± 1.60
Immunosuppressive treatment^e^, n (%)	4 (33.3)	7 (58.3)	8 (66.7)	19 (52.8)
**ILD**				
**Lung involvement in CT, n (%)**				
< 20%	N/A	11 (91.7)	8 (66.7)	19 (79.2)
≥ 20%	N/A	2 (11.1)	3 (25.0)	10 (27.8)
ILD disease duration^d^, years (mean ± SD)	N/A	1.7 ± 1.0	4.7 ± 2.7	3.2 ± 2.5
**Lung function parameters, % predicted (mean ± SD)**				
FVC% predicted (mean ± SD)	102.1 ± 18.8	108.1 ± 18.5	80.7 ± 22.8	96.9 ± 22.9
DLCO% predicted (mean ± SD)	79.2 ± 13.1	68.8 ± 17.7	58.6 ± 25.1	68.9 ± 20.6
FEV1% predicted (mean ± SD)	96.1 ± 19.2	98.3 ± 18.6	82.3 ± 20.7	92.2 ± 20.3
TLC% predicted (mean ± SD)	104.8 ± 18.6	108.9 ± 11.9	90.9 ± 23.6	101.5 (19.8)
FVC change, % (mean ± SD)	N/A	4.4 ± 7.1	− 14.5 ± 7.1	− 5.1 ± 11.9
DLCO change, % (mean ± SD)	N/A	2.1 ± 14.6	− 16.1 ± 21.6	− 6.6 ± 20.2
GAP index (mean ± SD)	N/A	1.2 ± 1.1	2.6 ± 2.1	1.2 ± 0.5
CPI index (mean ± SD)	N/A	22.4 ± 11.5	38.2 ± 20.8	30.3 ± 18.3

*BMI *body mass index, *CPI *composite physiologic index, *CRP *C-reactive protein, *dcSS *diffuse cutaneous SSc, *DLco *carbon dioxide diffusion capacity, *ESR *erythrocyte sedimentation rate, *FEV1 *forced expiratory volume in one second, *FVC *forced vital capacity, *lcSSc *limited cutaneous SSc, *GAP *gender-age-physiology, *mRSS *modified Rodnan skin score, *SD *standard deviation, *TLC *total lung capacity.

^a^n dependent on available datasets.

^b^Disease duration after onset of first non-Raynaud's symptoms, ^c^Symptoms reported by patients

^d^Disease duration from first diagnosis of SSc-ILD

^c^Including treatment with following medications: azathioprine, corticosteroids, hydroxychloroquine, leflunomide, methotrexate, mycophenolate mofetil, rituximab, and tocilizumab.

All SSc patients included in the study fulfilled the 2013 ACR/EULAR classification criteria^23^ at the time of blood withdrawal.

### Patients with progressing ILD show different clinical features compared with patients with stable ILD

**Figure 1:**
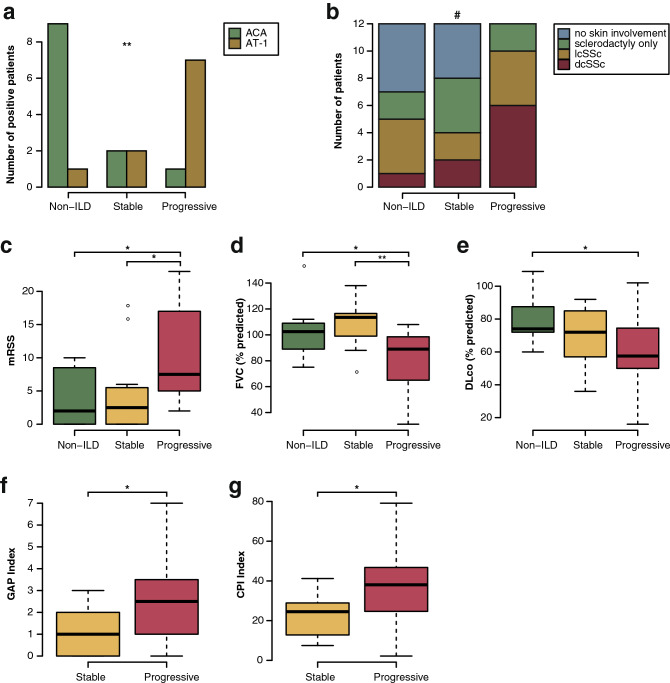
Clinical characteristics of SSc patients from the Zurich cohort at time point of sample collection. *mRSS *modified Rodnan skin score, *dcSSc *diffuse cutaneous SSc, *lcSSc *limited cutaneous SSc, *ACA *anti-Centromere antibodies, *AT-1 *anti-topoisomerase I antibodies, *FVC *forced vital capacity, *DLco *carbon monoxide diffusion capacity. *p ≤ 0.05, **p ≤ 0.01, ***p ≤ 0.001, ^#^not significant. N = 12 per group.

With respect to autoantibody profiles, a higher prevalence of anti-Centromere antibodies occurred in non-ILD patients (n = 9, 75%; p = 0.0045), of which the majority had only sclerodactyly or no skin involvement (n = 7, 58.3%) (Table 1). Anti-topoisomerase1-positivity, previously reported in the context of severe skin and lung involvement, was predominantly found in progressive SSc-ILD patients (n = 7, 58.3%, p = 0.0045, Fig. 1a) (Table 1, Fig. 1b).

While no difference was seen in time from onset from first non-Raynaud’s symptoms between the different patients’ groups, progressive patients had a significantly longer average duration of ILD than stable patients (mean ± SD = 4.7 ± 2.7 vs. 1.7 ± 1.0 years, p = 0.0028) and a more severe skin fibrosis as assessed by modified Rodnan skin score (mRSS) than both stable SSc-ILD and non-ILD patients (Table 1; Fig. 1c, mean ± SD = 10.3 ± 7.7 vs. 4.5 ± 5.8 and 3.7 ± 4.1, respectively; p = 0.024 and 0.011).

In addition, progressive SSc-ILD patients had a significantly lower forced vital capacity (FVC% predicted) than stable (mean ± SD = 80.7 ± 22.8 versus 108.1 ± 18.5 versus 102.1 ± 18.8, p = 0.0059 and p = 0.036, respectively) and non-ILD patients, while their diffusion capacity for carbon monoxide (DLco% predicted) was significantly lower than that of SSc patients without ILD (mean ± SD = 58.6 ± 25.1 versus 79.2 ± 13.1, p = 0.035) (Table 1, Fig. 1d,e). During the observation period, patients with progressive ILD experienced a significant decline in lung function compared with stable ILD patients (change FVC% predicted mean ± SD − 14.5 ± 7.1 versus 4.4 ± 7.1, p < 0.0001 and change DLco% predicted − 16.1 ± 21.6 versus 2.1 ± 14.6, p = 0.026; Table 1). Moreover, both the gender-age-physiology (GAP)^24^ and composite physiologic index (CPI)^25^ values, developed for the prediction of mortality in IPF patients, were higher in progressive than in stable SSc-ILD patients indicating a worse prognosis (mean ± SD = 2.6 ± 2.1 versus 1.2 ± 1.1 and 38.2 ± 20.8 versus 22.4 ± 11.5, p = 0.031 and 0.048, respectively, Table 1; Fig. 1f,g).

While there was a tendency towards a higher prevalence of immunosuppressive treatment, higher erythrocyte sedimentation rate (ESR) and higher C-reactive protein (CRP) levels in progressive SSc-ILD patients, changes were not statistically significant (Table 1). In addition, there was a non-significant tendency towards a lower body mass index (BMI) in progressive SSc-ILD compared to stable SSc-ILD and non-ILD patients (Table 1) (p = 0.35).

### Metabolic serum profiling detects differences between disease subtypes

To identify differentially regulated serum metabolites as potential discriminators between healthy individuals and different SSc subgroups, targeted metabolic profiling for 110 metabolites (Supplementary Table S1) using targeted LC–MS/MS analysis was performed. After data processing and filtration, a total of 85 serum metabolites was detected, 56 in ESI (electron spray ionization) positive, 24 in ESI negative mode, and 5 in both modes (Supplementary Table S2). To test our hypothesis of distinct and discriminating metabolite patterns we performed multivariate analysis (hierarchical clustering and PLS-DA), followed by univariate analysis. Performance of significant metabolites from both analyses was then assessed by ROC analysis.

**Figure 2:**
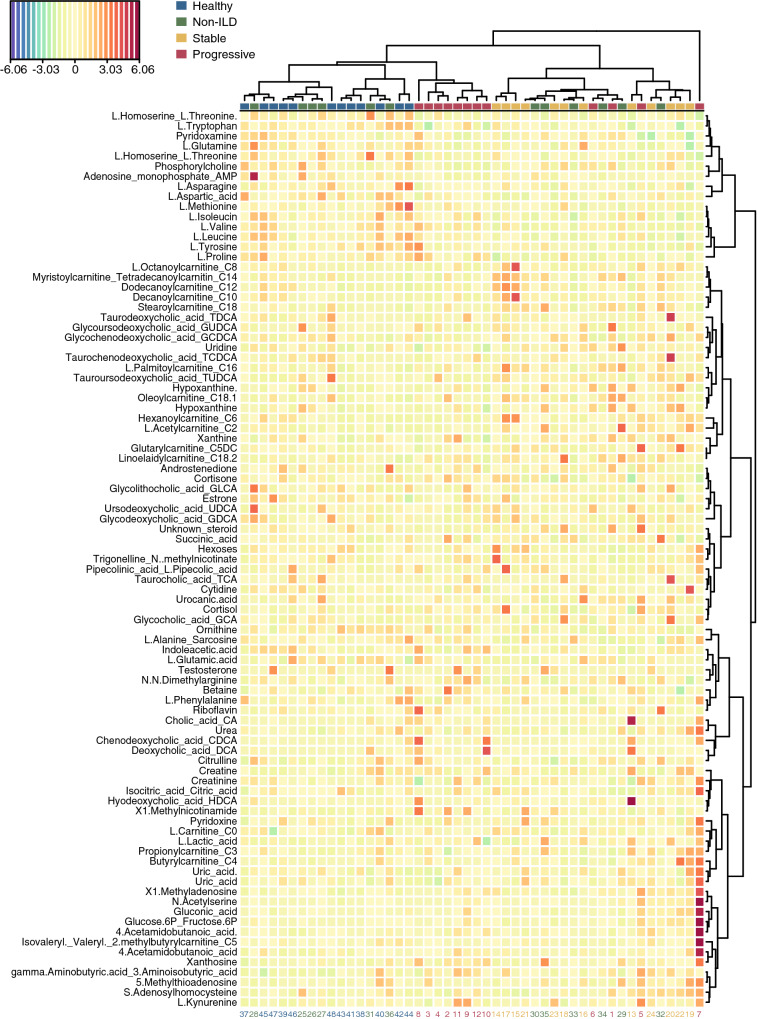
Hierarchical clustering of metabolomics data using Euclidean distance and direct linkage.

**Figure 3:**
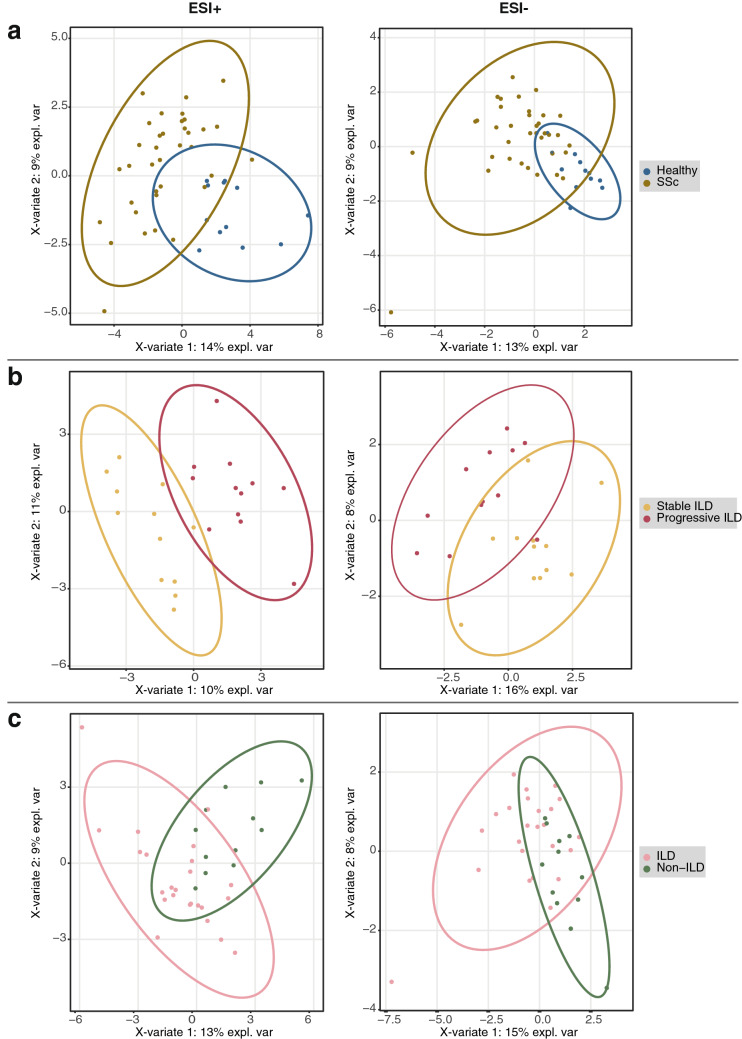
Group-wise PLS-DA analysis of metabolomics data. *ESI+/− *positive/negative electrospray ionization. (**a**) Healthy (n = 12) versus all SSc (n = 36); (**b**) stable versus progressive SSc-ILD (n = 12 per group); (**c**) all SSc-ILD (n = 24) versus non-ILD SSc (n = 12).

Hierarchical clustering of the targeted serum profiles suggested differences between the four groups (Fig. 2), which became more apparent in the subsequent PLS-DA (Fig. 3). Interestingly, the clearest separation was observed when comparing patients with progressing and stable ILD (Fig. 3b).

### Serum metabolites show potential as biomarkers

To define the best discriminating metabolites we used variable importance in projection (VIP) scores (Supplementary Tables S3a,b) with a rather strict threshold set at ≥ 2. Based on this approach, we found distinct metabolite signatures for the different group comparisons (Table 2). Healthy individuals and SSc patients were best distinguished by changes in the amino acids (AAs) l-tyrosine and l-tryptophan, whereas SSc patients with and without ILD were best classified by dysregulation of l-threonine, xanthosine, 3-aminoisobutyric acid, and adenosine monophosphate. Progressors compared to stable ILD patients were characterized by alterations of l-leucine, l-isoleucine, xanthosine, and adenosine monophosphate.

**Table 2:** Significant VIP scores from PLS-DA for all group comparisons by electrospray ionization (ESI) mode and x-variate component.

Metabolite	VIP scores		ESI mode	Groups
	Component 1	Component 2		
l-Tyrosine	2.05		Positive	Healthy vs. all SSc
l-Tryptophan	2.23	2.10		
l-Tryptophan	2.76	2.48	Negative	
l-Threonine	2.13		Positive	ILD vs. non-ILD SSc
3-Aminoisobutyric acid	2.06			
Adenosine monophosphate	2.48	2.03		
Xanthosine		2.11	Negative	
l-Isoleucine	2.14		Positive	Stable vs. progressive SSc-ILD
l-Leucine	2.41	2.21		
Adenosine monophosphate	2.01			
Xanthosine	2.75	2.09	Negative	

Significance is defined by VIP values ≥ 2 meaning that these metabolites were essential for the discrimination of different groups of patients in PLS-DA^14,21^.

In accordance with the results from the multivariate analysis, ANOVA-based univariate analysis with FDR correction for multiple testing identified l-leucine (p = 0.028), l-tyrosine (p = 0.077), xanthosine (p = 0.032), l-tryptophan (p = 0.028 for ESI+ and ESI− modes), and 1-methyladenosine (p = 0.077) as significantly altered between groups.

**Figure 4:**
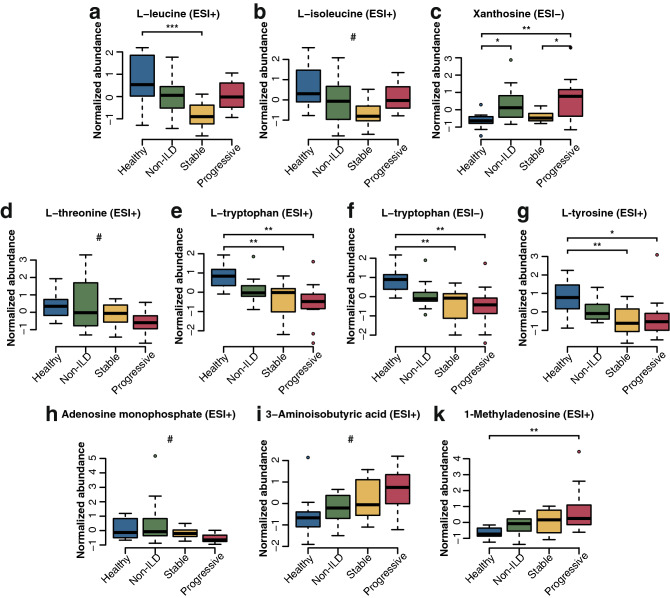
Significant metabolites in univariate analysis of Z-score-normalized peak areas and PLS-DA analysis. * = p ≤ 0.05, ** = p ≤ 0.01, *** = p ≤ 0.001, # = not significant in univariate analysis and no post-hoc test performed. N = 12 per group.

Analysis of peak areas showed that levels of l-leucine and l-isoleucine were highest in healthy individuals and gradually decreased from SSc patients without ILD to those with stable ILD. Interestingly, patients with progressing ILD had increased levels compared with stable SSc-ILD patients (Fig. 4a,b). For xanthosine, we observed significantly lower levels in healthy individuals compared with non-ILD SSc patients as well as for stable SSc-ILD patients compared with progressing SSc-ILD patients (Fig. 4c).

l-Tryptophan, l-tyrosine, l-threonine, and adenosine monophosphate showed a gradual decline from healthy individuals, SSc without ILD, stable ILD to progressive ILD (Fig. 4d–h), whereas the opposite occurred for 3-aminoisobutyric acid and 1-methyl-adenosine (Fig. 4i,j).

### Amino acid profiles discriminate between groups and correlate with changes in lung function and prognostic scores

**Figure 5:**
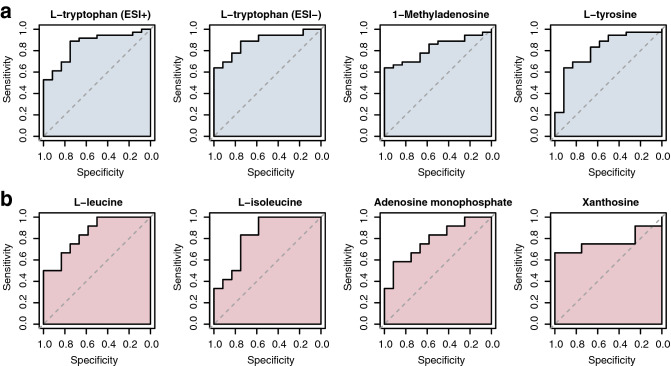
ROC analyses of significant metabolites for healthy (n = 12) versus all SSc (n = 36) (**a**) and stable (n = 12) versus progressive (n = 12) SSc-ILD (**b**). *AUC *area under the curve. Brackets show confidence intervals.

To select the best discriminators between two groups, we performed ROC curve analysis for the previously identified candidate serum metabolites.

l-Tryptophan, detected in both ESI+ and ESI− modes, best differentiated between healthy individuals and SSc patients (AUC 0.859, 95% CI 0.745–0.973; AUC 0.884, 95% CI 0.788–0.981), followed by 1-methyl-adenosine (AUC 0.822, 95% CI 0.705–0.939) and l-tyrosine (AUC 0.812, 95% CI 0.667–0.958) (Fig. 5a).

In the group of ILD patients, l-leucine and l-isoleucine performed best to distinguish progressing from stable SSc-ILD patients (AUC 0.847, 95% CI 0.695–1.00; AUC 0.826, 95% CI 0.656–0.997), followed by adenosine monophosphate (AUC 0.785, 95% CI 0.598–0.971), and xanthosine (AUC 0.771, 95% CI 0.551–0.990) (Fig. 5b). No significant performance of metabolites in separation of non-ILD SSc and SSc-ILD patients was observed.

**Figure 6:**
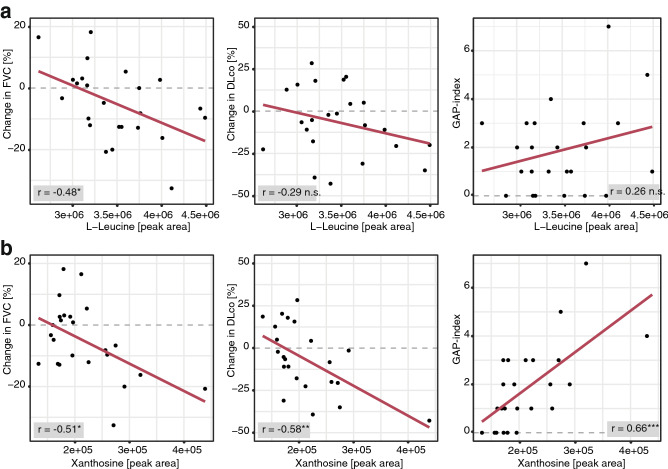
Pearson correlation analysis of l-leucine (**a**) and xanthosine (**b**) in SSc-ILD patients (n = 24). *p ≤ 0.05, **p ≤ 0.01, n.s. = p > 0.05.

Notably, in patients with SSc-ILD, both, l-leucine and xanthosine negatively correlated with changes in the lung function parameter FVC% predicted (r = − 0.48 and − 0.51; p = 0.016 and 0.011, respectively), while xanthosine also negatively correlated with changes in DLco% predicted (r = − 0.58; p = 0.0038) as well as with the GAP index (r = 0.67, p = 0.0005) (Fig. 6). The correlations with changes in absolute values of FVC and DLco% predicted are shown in Supplementary Fig. S1a,b.

**Figure 7:**
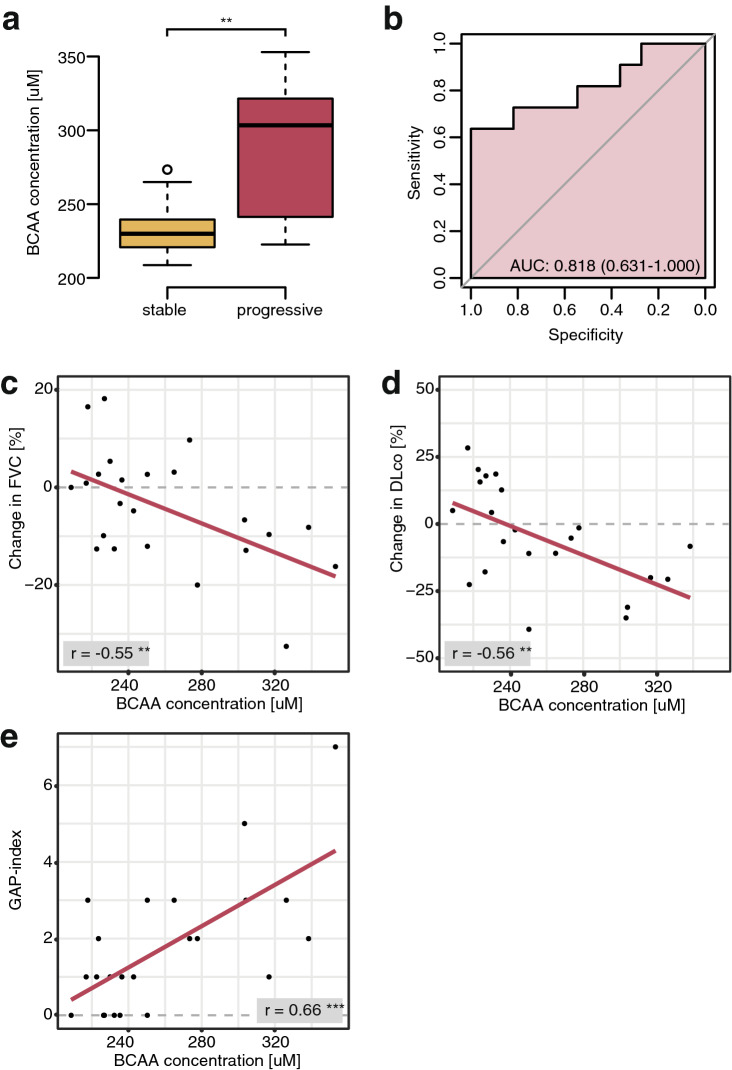
Results of t-test (**a**), ROC analysis (**b**), and Pearson’s correlation analysis (**c**,**d**) of enzymatic analysis of BCAA levels in SSc-ILD patients. **p ≤ 0.01. N = 11 (progressive) and 12 (stable).

Validation of higher values of the BCAAs l-leucine and l-isoleucine in progressive SSc-ILD compared to stable disease with the use of an enzymatic assay resulted in similar results as LC–MS/MS, with significantly higher values detected in progressive patients (mean = 286.5 and 235.5 µM, for progressive and stable patients, respectively; p = 0.005) (Fig. 7a). In ROC analysis (AUC 0.818, 95% CI 0.631–1.032), a cut-off value of 250.3 µM separated stable from progressive patients with a sensitivity of 72.7% and a specificity of 83.3% (Fig. 7b). Furthermore, BCAA levels negatively correlated with changes in FVC (r = − 0.55; p = 0.0063) and DLco% predicted (r = − 0.56; p = 0.0064) and positively with the GAP index (r = 0.66, p = 0.0005) (Fig. 7c–e). The correlations with changes in absolute values of FVC and DLco% predicted are shown in Supplementary Fig. S1c.

For external validation, we assessed BCAA levels in an independent, prospectively followed cohort of SSc-ILD patients from Paris. Herein, we found a similar trend towards higher BCAA levels in patients with future progression of ILD as compared with patients, who remained stable during follow-up (mean = 311.8 ± 26.5 and 289.6 ± 49.4 µM, respectively; p = 0.26). In ROC analysis, BCAA levels correlated negatively with DLco% predicted (r = − 0.38, p = 0.027), and positively with the mortality-predicting GAP index (r = 0.39, p = 0.022). The results are shown in Supplementary Fig. S2, for details including patients’ characteristics refer to data supplement.

In order to assess whether elevated BCAA levels specifically reflected fibrotic and/or pulmonary processes, we measured BCAA serum levels in 29 patients with non-fibrotic, primary myositis^26^ without concomitant ILD. Interestingly, BCAA levels significantly correlated with disease activity (Spearman’s rho = 0.52, p = 0.0037). Patients with CK levels exceeding the defined upper limit of normal (170 U/l) had significantly higher BCAA levels than patients with low CK levels (mean = 309.5 ± 88.2 and 253.4 ± 41.6 U/l for active and inactive disease, respectively; p = 0.032). No correlation with systemic inflammation, measured by CRP as a surrogate marker, was observed (Pearson’s r = − 0.15, p = 0.46). All results are visualized in Supplementary Fig. S3, for details including patients’ characteristics refer to data supplement.

## Discussion

Our study assessed the potential of serum metabolites as circulating biomarkers for disease stage and severity of SSc(-ILD).

Serum metabolite profiling yielded a final set of 4 amino acids and 3 purine metabolites. Changes in the levels of l-tyrosine, l-tryptophan, and 1-methyl-adenosine distinguished HC from SSc and alterations in l-leucine, l-isoleucine, xanthosine, and adenosine monophosphate profiles differentiated between progressing and stable SSc-ILD with l-tryptophan and l-leucine being the best performing discriminators in the respective groups. Increased serum levels of l-leucine and l-isoleucine in progressing compared with stable SSc-ILD patients were confirmed with an independent enzymatic assay with definition of a critical threshold.

Our results are in accordance with the previously reported changes in energy metabolism in fibrotic conditions^13–15^. Similar to our findings, previous studies in SSc and/or fibrosing ILD reported decreased serum levels of l-tryptophan^27,28^ or upregulation in IPF lungs^14^. Serotonin^29^, a downstream product of l-tryptophan, was upregulated in fibrotic conditions^30^ including SSc^31^. Inhibition of the serotonin receptors had anti-fibrotic effects in experimental conditions and in a first in human proof-of-concept study^31–34^. Elevated levels of 1-methyl-adenosine^29^ have so far been associated with proliferative and/or metastatic tumors^35,36^. Non-modified adenosine, generated extracellularly from ATP/ADP breakdown^29^, is a well-known pro-fibrotic mediator^37–41^ and its inhibition was beneficial in in fibrotic animal models^37,39,40^. As previously observed in lung fibrosis and SSc^14^, there was a tendency towards lower adenosine monophosphate (AMP) levels in progressing SSc-ILD patients. Interestingly, AMP-activated protein kinase (AMPK), a critical sensor of energy sufficiency, acts as central metabolic switch in cell metabolism and thereby opposes mTOR (mechanistic target of rapamycin) signaling^42^. Activation of AMPK causes a shift from anabolism to catabolism to generate ATP to restore energy homeostasis. In fibrotic conditions, AMPK activity was decreased^43^. Activation of AMPK reversed established lung fibrosis in the bleomycin-induced lung fibrosis model^16,44^. Patients with progressing compared with stable ILD displayed increased levels of l-leucine and l-isoleucine. Both essential AA are highly abundant in elastin^45^, a major extracellular matrix (ECM) protein, and could thus reflect increased ECM degradation ^46^. Both AAs were upregulated in the lung tissue and the exhaled breath condensate of patients with IPF^14,19^. BCAAs, particularly l-leucine, stimulate protein synthesis and reduce protein breakdown via the phosphorylation of mTOR^47^. mTOR plays an important role in anabolic processes by causing cells to switch from oxidative phosphorylation to aerobic glycolysis^48^. In SSc und pulmonary fibrosis, mTOR activity is increased^49^. Its inhibition by rapamycin prevented experimental fibrosis and showed some benefit in diffuse cutaneous SSc patients in a small, randomized, phase 1 study^48^. Finally, levels of xanthosine, a purine nucleoside, were higher in progressing compared with stable SSc-ILD. Purine receptors were suggested to play a role in fibrosis^50^.

Clinically, in our study, progressive SSc-ILD patients were characterized by a higher prevalence of anti-topoisomerase 1 antibodies, more severe skin fibrosis, worse lung function and worse prognostic scores. In addition, they showed a substantial decline of lung function in the observation period. Of note, both l-leucine and xanthosine negatively correlated with pulmonary function (changes in FVC% and DLco% predicted respectively) and one ILD-mortality prediction score (GAP index), which underlines their relevance as biomarker candidates. Most importantly, we could validate l-leucine and l-isoleucine, the best discriminators of progressing vs. stable SSc-ILD, in an independent experiment. In the BCAA assay, a defined cut-off value of 250.3 µM distinguished stable from progressive patients with good sensitivity and specificity and again BCAA levels negatively correlated with changes of lung function parameters and positively with the GAP index. Similar results were obtained by analysing an independent, external validation cohort of SSc-ILD patients from Paris.

The limitations of our study mainly arise from the limited number of patients. Validation in external multi-centre cohorts will be needed to assess the future usefulness in clinical routine. Furthermore, potential correlations with other protein biomarkers should be assessed. Prediction modelling for progression of ILD with circulating biomarkers, clinical, functional, and imaging parameters would be ideal to test the performance of circulating biomarkers only models versus mixed models. This, however, again warrants large datasets and multi-centre cohorts.

Disturbances in AA metabolism have been reported in other studies on SSc and IPF. Differences in identified metabolite profiles might arise from different analysis and detection methodologies (i.e. mass spectrometry or ion exchange chromatography) or differences in sample collection, storage and processing. We decided on a large-scale targeted analysis on a triple quadrupole mass spectrometer rather than an untargeted full-scan approach applying high-resolution mass spectrometry due to the increased sensitivity, linearity, reproducibility and straight-forward metabolite identification of targeted LC–MS/MS acquisition^51^. Although the applied assay covered multiple differentially regulated pathways, it is limited to the tested 110 metabolites. Further research could be carried out using untargeted metabolic profiling or a targeted assay covering a wider range of metabolites such as carbohydrates or phospholipids.

Additionally, given the real-life scenario, we cannot exclude that the fasting state and/or the diet might have some influence especially on the measured AA levels. Supplementation with micronutrients and/or vitamins did not occur in our patients’ cohort and we draw blood at approximately the same time in the morning to eliminate potentially confounding factors as well as possible. The fact, however, that changes in (BC)AA levels were reported consistently in other studies of lung fibrosis argues against a strong or exclusively dietary influence.

Furthermore, we have to take into account that SSc is a multi-organ disease. In our study, patients with progressive ILD also had more extensive skin disease pointing towards a more severe disease state. We can therefore not assume that changes in BCAA serum levels exclusively reflect lung pathology. Taking into account the correlation between CK and BCAA levels in primary myositis patients, it seems likely that in both myositis and SSc(-ILD), high serum BCAA levels reflect overall disease activity characterized by a switch to an anabolic state with subsequent changes in AA metabolism. Thus, in these complex diseases, changes in BCAA levels can probably not be attributed to either pro-fibrotic or immune processes since they may rather reflect the disturbed metabolism that arises from global tissue remodelling with varying contributions of different cell types. This argues, however, not against the usefulness of BCAA as progression markers in a given disease context.

In conclusion, our study suggests that serum metabolites might have potential as circulating biomarkers for discriminating stable and progressive SSc patients. Confirmation in larger multi-cohorts will be needed to fully appreciate their value for routine clinical care.

## Methods

### Patients and controls

For this study, SSc patients from the University Hospital Zurich’s prospective SSc patients cohort were divided in the following three groups: patients without ILD (non-ILD), patients with stable ILD and patients with progressive ILD (n = 12 per group).

Progressive ILD was defined as either a relative decrease in FVC% predicted of ≥ 10% independent of changes in DLco% predicted, a decrease in FVC% predicted of 5–9% combined with a decrease in DLco% predicted of ≥ 15%, or an increase of pathologic lung involvement in high resolution computed tomography (HRCT) from < 20% to > 20% compared to the previous visit [mean follow-up interval = 14 months (range = 9–26)]^52,53^. Stable ILD was defined as the absence of the above-mentioned criteria for progression in any of the visits recorded in the EUSTAR database^54^. Non-ILD patients were defined as having no evidence of lung involvement on HRCT scans.

Progressive SSc-ILD patients were matched with stable SSc-ILD and non-ILD SSc patients as well as healthy controls (HC, n = 12) for age, sex, and time point of blood withdrawal (morning).

In addition, serum BCAA levels were analyzed in two additional cohorts of SSc-ILD and primary myositis patients. Detailed information on these patients can be found in the Supplementary Methods, data supplement pp. 15.

Serum collection and processing was performed following a standardized protocol in accordance with international guidelines^55^. Aliquots of serum were stored at − 80 °C until further processing.

Written informed consent was obtained from all enrolled individuals.

The study was approved by the canton of Zurich’s ethics committee (approval numbers: pre-BASEC-EK-839 (KEK-no.-2016-01515), KEK-ZH-no. 2010-158/5, BASEC-no. 2018-01873, BASEC-no. 2018-02165, BASEC-no. 2017-01298, KEK-ZH-Nr. 2012-0419) and all experiments were conducted in accordance with Swiss legislation and regulation.

### Ultra-high performance liquid chromatography coupled to tandem mass spectrometry (UHPLC-MS/MS)

Serum profiling of 110 metabolites of SSc patients and HC was performed using a targeted ultra-high performance liquid chromatography coupled to tandem mass spectrometry (LC–MS/MS) assay as described previously^56^.

For metabolite extraction, frozen serum samples were thawed at room temperature and 300 μL of ice-cold acetonitrile:ethanol (1:1) were added to 100 μL of each sample. Samples as well as an equally treated water-only control were vortexed, incubated at − 80 °C for 30 min, centrifuged at 14,000*g* and 4 °C for 15 min in order to pellet precipitated proteins. 350 μL of supernatant of each sample were transferred to fresh tubes and dried using a speed vacuum centrifuge at room temperature and minimum pressure 5.1 Torr (Savant SPD1010, Thermo). Dried samples were stored at − 80 °C until further processing. Before data acquisition, samples were reconstituted in 300 μL 10% methanol, resulting in a final dilution of 1:3 in respect to the initial serum volume, sonicated for 1 min in a water bath in order to ensure complete reconstitution, and centrifuged at 14,000*g* and 4 °C for 20 min. Supernatants were transferred into glass vials and a quality control (QC) sample was generated by pooling 10 µL of all extracted samples. After, samples were stored at 10 °C until analysis.

Extracted samples were analysed on a Xevo TQ-S triple quadrupole mass spectrometer interfaced with an electrospray ionization source and coupled to an ACQUITY UPLC I-Class system (both Waters, USA). Chromatographic separation of 1 µL extract was performed using reversed-phase chromatography (ACQUITY UPLC HSS T3, 2.1 × 100 mm, 1.8 µm column, Waters, USA) with mobile phases composed of (A) 0.1% formic acid in H_2_O and (B) 0.1% formic acid in methanol. Further LC conditions and MS-specific parameters were previously described^56^. LC–MS/MS analysis was performed in negative electrospray ionization (ESI−) and positive electrospray ionization (ESI+) mode by two independent injections.

Before analysis of the first sample, the instrument was equilibrated by injecting the QC sample 10 times. Samples were then injected in a randomized block design order with intermittent analysis of the QC sample after every fifth sample in order to observe instrumental fluctuations.

### Branched chain amino acid assay

For quantification of the branched chain amino acids (BCAAs) leucine, isoleucine and valine, a commercial colorimetric analysis kit (ab83374, Abcam, United Kingdom) was used following the manufacturer’s instructions. Briefly, leucine standards and 2.5 times diluted serum samples were incubated for 30 min at room temperature with an equal volume of enzyme and substrate mix, initiating the colorimetric reaction. Absorption at 450 nm was measured using a GloMax-Multi Detection System microplate reader (Promega, USA) and sample BCAA concentration was calculated in relation to the standard curve.

### Data analysis

Raw metabolomics data were processed in Skyline 4.2 (MacCoss Lab Software, USA). Metabolites were selected for statistical analysis if the peak area decreased linearly in diluted samples, no detection of background noise in extracted blank sample was observed, the coefficient of variation was ≤ 20% in QC samples, as well as, a proper peak shape was detected. Peak areas were then used for further statistical analysis as specified below.

Statistical data analysis and graphical visualization was performed using R 3.6 with the mixOmics package and GraphPad Prism 8.0.0 (GraphPad Software, USA).

For univariate analysis and hierarchical clustering, metabolite peak areas were Z-score transformed for normalization.

Univariate analysis of metabolomics data was performed by applying one-way ANOVA as well as Tukey’s post-hoc test for multi-group data. Two-group data was analyzed by Student’s t-test or Mann–Whitney U test for parametric and nonparametric data, respectively. Categorical data were analysed using a Chi-Square test. Performance of potential biomarkers was assessed by Receiver Operating Characteristic (ROC) curve analysis. Pearson’s (parametric) and Spearman’s (non-parametric) correlation was used to assess linear relationships between metabolites and clinical parameters. For multivariate analysis, data were subjected to partial least-square discriminant analysis (PLS-DA) with variable importance in projection (VIP) scores of ≥ 2 being considered statistically significant.

Data are presented either as medians with interquartile range (boxplots; horizontal line = median, boxes = interquartile range) or as means with standard deviation (SD; tables). For false discovery rate (FDR)-corrected univariate analysis of metabolites (excluding post-hoc testing), the significance level was set to 0.1, while for all other analyses p-values < 0.05 were considered statistically significant.

## Supplementary Information

Supplementary Information.

## Data Availability

All data are presented either in the main text or in the data supplement. The datasets generated and/or analysed during the current study are available from the corresponding author on reasonable request.
