# Supplementary material for: Serum metabolites as biomarkers in systemic sclerosis-associated interstitial lung disease
Source: Sci Rep. 2020 Dec 14;10:21912. doi: 10.1038/s41598-020-78951-6 (PMC7736572; doi:10.1038/s41598-020-78951-6)
Supplement: Supplementary file 1 — Supplementary Information. [file 41598_2020_78951_MOESM1_ESM.docx]

**Serum Metabolites as Biomarkers in Systemic Sclerosis-Associated Interstitial Lung Disease – Data Supplement**

Meier C^1^, Freiburghaus K^2^, Bovet C^2^, Schniering J^1^, Allanore Y^3^, Distler O^1^, Nakas C^2,4^, Maurer B^1*^

^1^Center of Experimental Rheumatology, Department of Rheumatology, University Hospital Zurich, Switzerland

^2^University Institute of Clinical Chemistry, Bern University Hospital, University of Bern, Switzerland

^3^Department of Rheumatology A, Descartes University, APHP, Cochin Hospital, Paris, France

^4^Laboratory of Biometry, University of Thessaly, Volos, Greece

* Corresponding author: e-mail: [Britta.maurer@usz.ch](mailto:Britta.maurer@usz.ch); phone: +41 44 255 21 16; fax: +41 44 255 78 89

**Table of Contents**

Supplementary Results 3

Supplementary Table S1 3

Supplementary Table S2 6

Supplementary Table S3a 7

Supplementary Table S3b 8

Supplementary Table S4 10

Supplementary Table S5 11

Supplementary Figure S1 12

Supplementary Figure S2 13

Supplementary Figure S3 14

Supplementary Methods 15

**Supplementary Table S1:** Metabolites measured by LC-MS/MS (continued on next pages).


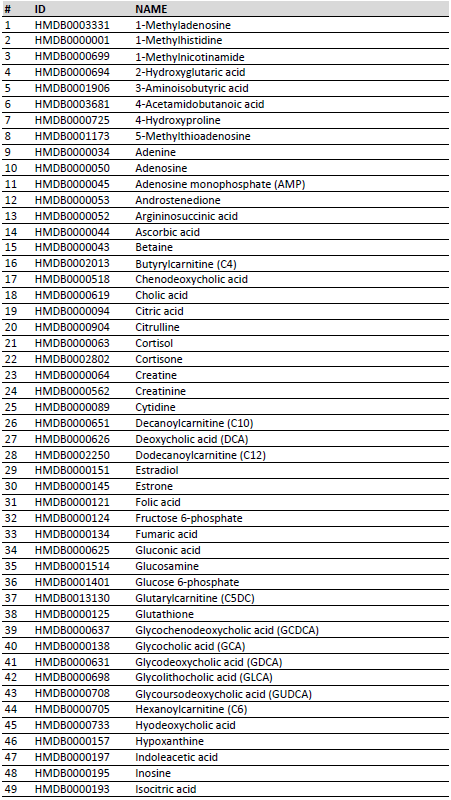


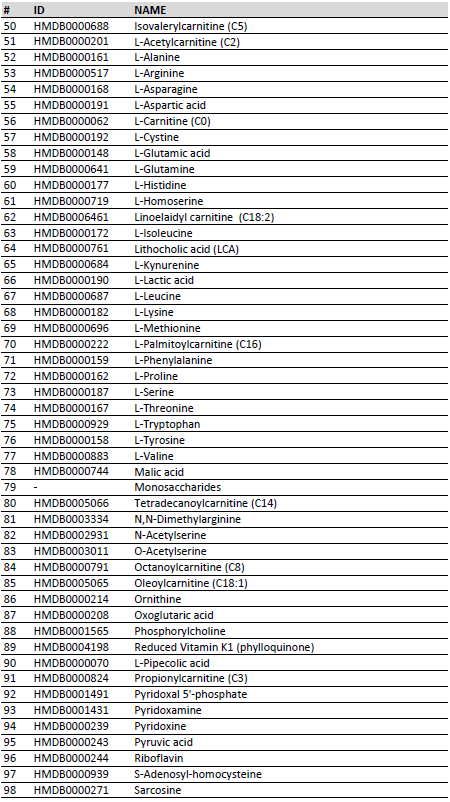


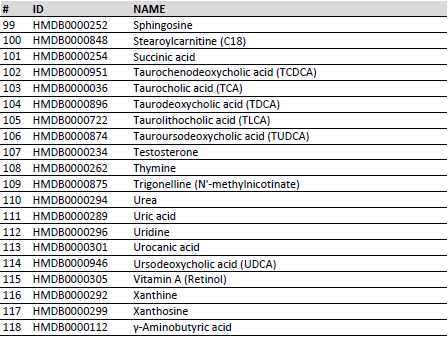


**Supplementary Table S2:** Metabolites detected by LC-MS/MS. ESI=electrospray ionization mode, + =positive, -=negative.

| **ESI+ (n = 56)** | **ESI- (n = 24)** | **Both (n = 5)** |
| --- | --- | --- |
| 1-Methyladenosine | Chenodeoxycholic acid | 4-Acetamidobutanoic acid |
| 1-Methylnicotinamide | Cholic acid | Hypoxanthine |
| 5'-Methylthioadenosine | Deoxycholic acid | L-Homoserine/L-Threonine |
| Adenosine monophosphate | Gluconic acid | L-Tryptophan |
| Androstenedione | Glucose-6P/Fructose-6P | Uric acid |
| Betaine | Glycochenodeoxycholic acid |  |
| Butyrylcarnitine | Glycocholic acid |  |
| Citrulline | Glycodeoxycholic acid |  |
| Cortisol | Glycolithocholic acid |  |
| Cortisone | Glycoursodeoxycholic acid |  |
| Creatine | Hexoses |  |
| Creatinine | Hyodeoxycholic acid |  |
| Cytidine | Isocitric acid/citric acid |  |
| Decanoylcarnitine | L-Aspartic acid |  |
| Dodecanoylcarnitine | L-Lactic acid |  |
| Estrone | Succinic acid |  |
| gamma-Aminobutyric acid | Taurochenodeoxycholic acid |  |
| Glutarylcarnitine | Taurocholic acid |  |
| Hexanoylcarnitine | Taurodeoxycholic acid |  |
| Indoleacetic acid | Tauroursodeoxycholic acid |  |
| Isovaleryl-Valeryl-2-methylbutyrylcarnitine | Uridine |  |
| L-Acetylcarnitine | Ursodeoxycholic acid |  |
| L-Alanine/Sarcosine | Xanthine |  |
| L-Asparagine | Xanthosine |  |
| L-Carnitine |  |  |
| L-Glutamic acid |  |  |
| L-Glutamine |  |  |
| Linoelaidylcarnitine |  |  |
| L-Isoleucin |  |  |
| L-Kynurenine |  |  |
| L-Leucine |  |  |
| L-Methionine |  |  |
| L-Octanoylcarnitine |  |  |
| L-Palmitoylcarnitine |  |  |
| L-Phenylalanine |  |  |
| L-Proline |  |  |
| L-Tyrosine |  |  |
| L-Valine |  |  |
| Myristoylcarnitine/Tetradecanoylcarnitin |  |  |
| N,N-Dimethylarginine |  |  |
| N-Acetylserine |  |  |
| Oleoylcarnitine |  |  |
| Ornithine |  |  |
| Phosphorylcholine |  |  |
| Pipecolinic acid/L-Pipecolic acid |  |  |
| Propionylcarnitine |  |  |
| Pyridoxamine |  |  |
| Pyridoxine |  |  |
| Riboflavin |  |  |
| S-Adenosylhomocysteine |  |  |
| Stearoylcarnitine |  |  |
| Testosterone |  |  |
| Trigonelline/N'-methylnicotinate |  |  |
| Unknown steroid |  |  |
| Urea |  |  |
| Urocanic acid |  |  |

**Supplementary Table S3a: VIP scores of metabolites detected in ESI- LC-MS/MS for different** PLS-DA group comparisons. Significant scores (≥2) are marked in red.

|  | **healthy vs. SSc-ILD** | | **non-ILD SSc vs. SSc-ILD** | | **stable vs. Progressive SSc-ILD** | |  |
| --- | --- | --- | --- | --- | --- | --- | --- |
| **Metabolite** | **component 1** | **component 2** | **component 1** | **component 2** | **component 1** | **component 2** |  |
| Cholic.acid | 0.591881989 | 1.30934268 | 1.314576881 | 1.11496875 | 0.31518661 | 1.338669857 | **ESI -** |
| Glycocholic.Acid | 0.453107948 | 0.49945872 | 0.955758792 | 0.749874182 | 0.482030822 | 0.364225077 |  |
| Taurocholic.Acid | 0.406995645 | 0.362992138 | 0.273254787 | 0.240106516 | 0.624067182 | 0.622669082 |  |
| Glycolithocholic.acid | 0.008209178 | 0.33224588 | 0.269005236 | 0.390940384 | 0.347042086 | 1.115156346 |  |
| Deoxycholic.Acid | 0.942179583 | 0.821533454 | 1.15306704 | 0.921122556 | 1.181681115 | 1.028303538 |  |
| Chenodeoxycholic.Acid | 0.713581408 | 1.183437047 | 1.564873416 | 1.387282808 | 1.630015734 | 1.338981455 |  |
| Hyodeoxycholic.Acid | 1.10439669 | 0.976305901 | 0.47644332 | 0.425189119 | 0.447800886 | 1.263480409 |  |
| Ursodeoxycholic.Acid | 1.123967739 | 1.275345575 | 0.166294902 | 0.20431567 | 0.325106675 | 0.294124921 |  |
| Taurodeoxycholic.Acid | 0.133381167 | 0.113607299 | 0.588559266 | 0.606496075 | 1.004646203 | 1.088516039 |  |
| Taurochenodeoxycholic.Acid | 0.328444853 | 0.306379067 | 0.389671821 | 0.345924196 | 1.065598331 | 0.843807432 |  |
| Tauroursodeoxycholic.Acid | 0.550224136 | 0.498963473 | 0.181319987 | 0.257392766 | 1.50053512 | 1.096799193 |  |
| Glycodeoxycholic.acid | 0.440143137 | 0.370312314 | 0.564593316 | 0.446097516 | 0.740734605 | 0.774653843 |  |
| Glycochenodeoxycholic.acid | 0.01415012 | 0.366205551 | 1.377451677 | 1.091698853 | 1.178358034 | 0.867785883 |  |
| Glycoursodeoxycholic.Acid | 0.490781478 | 0.478797352 | 0.554731051 | 0.560978259 | 0.391320828 | 0.520475164 |  |
| Hypoxanthine. | 1.682242618 | 1.528536845 | 0.542895328 | 0.561366272 | 0.628495268 | 0.460417235 |  |
| Xanthine | 0.946046049 | 0.796235205 | 0.296818848 | 0.717412417 | 0.150224339 | 0.512225695 |  |
| Uric.acid. | 0.815686301 | 0.836637791 | 0.072240047 | 0.95535558 | 0.63638257 | 1.166169722 |  |
| Xanthosine | 1.962749487 | 1.655927391 | 0.734701278 | 2.10786721 | 2.748862056 | 2.091345665 |  |
| L.Tryptophan. | 2.759249514 | 2.478980675 | 1.717166472 | 1.456084195 | 0.170904859 | 1.28795603 |  |
| L.Threonine | 1.030068611 | 1.091433504 | 1.923519898 | 1.812480179 | 1.308707347 | 1.003567672 |  |
| L.Aspartic.acid | 1.304930062 | 1.206116445 | 1.756037733 | 1.43351127 | 1.545794547 | 1.719118364 |  |
| Gluconic.acid | 1.029174231 | 1.092068881 | 1.26594589 | 1.235560559 | 0.86155826 | 0.679131701 |  |
| Uridine | 0.871238887 | 0.781807673 | 1.047871541 | 0.97828883 | 0.542434997 | 0.697166757 |  |
| L.Lactic.acid | 0.767350474 | 0.904040757 | 0.326870015 | 0.303381787 | 0.335778858 | 0.260389559 |  |
| D.Glucose | 0.250874688 | 0.220545404 | 0.501880072 | 0.400696621 | 1.13074388 | 1.074062462 |  |
| Glucose.6.phosphate | 0.934420269 | 1.10187878 | 1.187049646 | 1.258311081 | 1.102927043 | 0.807518458 |  |
| Succinic.acid | 0.321540507 | 0.383586858 | 1.218269121 | 0.99703569 | 0.042151216 | 0.430730519 |  |
| Citric.acid | 0.631538259 | 0.926741962 | 1.218882136 | 0.975827395 | 0.317108836 | 0.801774858 |  |
| X4.Acetamidobutanoic.acid. | 1.034479295 | 1.090417947 | 1.012386711 | 1.348137321 | 1.027260298 | 0.841690278 |  |

**Supplementary Table S3b**: VIP scores of metabolites detected in ESI+ LC-MS/MS for different PLS-DA group comparisons. Significant scores (≥2) are marked in red.

|  | **healthy vs. SSc-ILD** | | **non-ILD SSc vs. SSc-ILD** | | **stable vs. Progressive SSc-ILD** | |  |
| --- | --- | --- | --- | --- | --- | --- | --- |
| **Metabolite** | **component 1** | **component 2** | **component 1** | **component 2** | **component 1** | **component 2** |  |
| Linoelaidyl.carnitine | 1.258984702 | 1.057269847 | 0.380154605 | 0.326730376 | 0.642336525 | 0.831971758 | **ESI +** |
| Butyrylcarnitine | 0.307138493 | 1.017592317 | 1.377077304 | 1.470016714 | 0.636403755 | 0.589565021 |  |
| L.Carnitine | 0.707228765 | 0.651662118 | 0.825678884 | 0.64060537 | 0.33998558 | 0.414990157 |  |
| Decanoylcarnitine | 0.535488008 | 0.613087225 | 0.575250045 | 0.448215248 | 1.276637335 | 1.320340376 |  |
| Dodecanoylcarnitine | 0.512677653 | 0.822461306 | 0.462301668 | 0.36272245 | 1.456072734 | 1.382834782 |  |
| Hexanoylcarnitine | 0.353468732 | 1.152015633 | 1.131104047 | 0.995700292 | 1.548310383 | 1.472070324 |  |
| Isovalerylcarnitine | 0.240090516 | 0.983281633 | 0.939728856 | 1.147751625 | 0.364976445 | 0.355652886 |  |
| Myristoylcarnitine | 0.716085624 | 0.903958976 | 0.103673821 | 0.239923315 | 1.284773809 | 1.261898511 |  |
| L.Acetylcarnitine | 0.765130064 | 1.097542953 | 0.150774029 | 1.37433793 | 1.688821561 | 1.57165552 |  |
| L.Octanoylcarnitine | 0.73923317 | 0.752517392 | 0.854949674 | 0.665654002 | 1.374787583 | 1.381727932 |  |
| Oleoylcarnitine | 0.919063545 | 0.824606297 | 0.602312318 | 0.821320895 | 0.333202208 | 0.483301297 |  |
| L.Palmitoylcarnitine | 0.761215915 | 0.80798719 | 0.111845937 | 0.169355859 | 0.649065947 | 0.80877253 |  |
| Propionylcarnitine | 0.084386962 | 0.836752373 | 1.274576311 | 1.276901278 | 1.0289706 | 0.979051024 |  |
| Stearoylcarnitine | 0.855518356 | 0.787943233 | 0.462803075 | 0.376101526 | 0.984092356 | 0.962044403 |  |
| Glutarylcarnitine | 0.614556299 | 0.794756787 | 0.600258137 | 0.849494503 | 0.64686966 | 0.68778421 |  |
| L.Alanine | 0.462452608 | 0.734786047 | 0.019756246 | 0.106638466 | 0.514280906 | 0.504060338 |  |
| L.Methionine | 1.593503011 | 1.3473257 | 0.493524282 | 0.60286236 | 1.048901203 | 0.957331097 |  |
| S.Adenosylhomocysteine | 1.386865457 | 1.166097991 | 0.232726444 | 0.85014628 | 0.344240476 | 0.330854203 |  |
| Creatine | 0.528282973 | 0.702949139 | 1.603566755 | 1.257097734 | 0.10510967 | 0.221718025 |  |
| Creatinine | 0.137282343 | 0.828820503 | 0.016541893 | 1.053284985 | 0.133466052 | 0.125050544 |  |
| N.N.Dimethylarginine | 0.241931695 | 0.242709401 | 0.838930369 | 0.655520658 | 1.513654283 | 1.486355923 |  |
| L.Threonine | 0.841888676 | 1.029410092 | 2.129941121 | 1.678754055 | 1.33998023 | 1.318042369 |  |
| L.Proline | 1.320071295 | 1.330275269 | 0.140076651 | 0.92336705 | 1.690396853 | 1.637186259 |  |
| X3.Aminoisobutyric.acid | 1.503777013 | 1.266323763 | 2.062327489 | 1.670075845 | 1.030265777 | 1.110588292 |  |
| L.Glutamine | 0.916366314 | 1.324215893 | 1.503158152 | 1.350402556 | 0.102237079 | 0.311550151 |  |
| L.Glutamic.acid | 0.309156972 | 0.26161476 | 0.27441992 | 0.799002152 | 0.503642816 | 0.837709635 |  |
| L.Isoleucine | 1.704224961 | 1.429111151 | 0.662914796 | 0.622788827 | 2.143535505 | 1.976880799 |  |
| L.Leucine | 1.964351926 | 1.654073594 | 1.357831709 | 1.061213872 | 2.405015971 | 2.209421172 |  |
| L.Valine | 1.542328913 | 1.291173721 | 0.540973539 | 0.627327784 | 1.267040555 | 1.161466099 |  |
| L.Pipecolic.acid | 0.203027002 | 0.432075556 | 0.219988693 | 0.470644128 | 0.336556899 | 0.399011773 |  |
| Ornithine | 1.184324345 | 1.048548285 | 1.242474477 | 0.989173242 | 0.820344329 | 0.749604046 |  |
| Citrulline | 0.406524566 | 0.666227675 | 0.263592184 | 0.214551185 | 1.932016707 | 1.76119273 |  |
| Urea | 0.547370677 | 1.263762517 | 0.462221532 | 1.457824419 | 0.814754152 | 0.813254661 |  |
| X4.Acetamidobutanoic.acid | 0.863280905 | 0.728447799 | 1.455104891 | 1.180621721 | 0.931076224 | 0.845246662 |  |
| L.Phenylalanine | 0.786304602 | 0.733375671 | 0.516850513 | 0.888486973 | 0.85617815 | 0.869724043 |  |
| L.Tyrosine | 2.045756 | 1.716299414 | 1.516379731 | 1.188588355 | 0.40648078 | 0.854266176 |  |
| Betaine | 0.199870821 | 0.184013809 | 1.33522154 | 1.591344946 | 1.141718483 | 1.109917729 |  |
| N..methylnicotinate | 0.409138307 | 0.589335068 | 0.818003264 | 0.643220073 | 0.477888873 | 0.443853718 |  |
| L.Tryptophan | 2.234723183 | 2.100613376 | 1.820986044 | 1.52654503 | 0.299413365 | 0.300730544 |  |
| Indoleacetic.acid | 0.670853936 | 0.584443391 | 0.973037253 | 1.277849035 | 0.536026844 | 0.73302284 |  |
| L.Kynurenine | 1.562590954 | 1.324345962 | 0.998894611 | 0.823766518 | 1.029835701 | 0.96449734 |  |
| Urocanic.acid | 0.871387161 | 0.729393255 | 0.214601604 | 0.388543267 | 0.104188168 | 0.164536115 |  |
| Phosphorylcholine | 0.947137334 | 0.800276936 | 1.22737092 | 1.098664381 | 0.956425669 | 0.869215395 |  |
| Riboflavin | 0.077788838 | 0.359672576 | 0.333490223 | 0.262827462 | 0.844477279 | 0.909726572 |  |
| Hypoxanthine | 1.403633234 | 1.307116367 | 0.613342123 | 0.515388364 | 0.722004459 | 0.683363158 |  |
| Uric.acid | 0.729283072 | 0.906411271 | 0.069288184 | 0.890214079 | 0.433093206 | 0.537759227 |  |
| X5..Methylthioadenosine | 0.783407625 | 0.842396588 | 0.230203479 | 1.640155629 | 0.038547187 | 0.069489833 |  |
| X1.Methyladenosine | 1.728670872 | 1.44632928 | 1.528434493 | 1.238255836 | 1.11488683 | 1.201543995 |  |
| Adenosine.monophosphate | 0.299519598 | 0.795506513 | 2.480738338 | 2.02751301 | 2.006007102 | 1.841596925 |  |
| Androstenedione | 0.11670789 | 0.147860873 | 0.741220635 | 0.735392481 | 0.607980877 | 0.687019191 |  |
| Cortisol | 1.259974402 | 1.182395211 | 0.620498588 | 0.662478104 | 0.493335169 | 0.874861262 |  |
| Cortisone | 0.274117892 | 0.263754607 | 0.179048634 | 0.615696111 | 1.007100648 | 1.215108522 |  |
| Testosterone | 0.05812792 | 0.062097686 | 0.538227476 | 0.457082959 | 0.55636792 | 0.701926397 |  |
| Estrone | 0.450640338 | 0.49070542 | 1.229195348 | 0.985346952 | 0.100526264 | 0.300489568 |  |
| X1.Methylnicotinamide | 1.507249724 | 1.705861865 | 0.233446083 | 0.71418372 | 0.808974238 | 0.827798925 |  |
| Cytidine | 0.175358944 | 0.239317595 | 0.298267636 | 0.405975188 | 0.727950441 | 1.030730939 |  |
| L.Asparagine | 1.461582996 | 1.273900384 | 1.117130725 | 0.955278173 | 0.085061379 | 0.81900729 |  |
| Pyridoxine | 0.733099852 | 0.702192904 | 0.256882967 | 0.802697415 | 0.138641369 | 0.209610504 |  |
| Pyridoxamine | 1.162039332 | 1.012957645 | 1.944587119 | 1.519985313 | 0.675586796 | 0.613977556 |  |
| N.Acetylserine | 1.209957062 | 1.098549268 | 1.035542991 | 1.261971879 | 0.70852248 | 0.682314314 |  |

.

**Supplementary Table S4:** Clinical characteristics of SSc-ILD patients of the Cochin Hospital Paris at the time of sample collection. mRSS = modified Rodnan skin score, dcSSc = diffuse cutaneous SSc, lcSSc = limited cutaneous SSc, FVC = forced vital capacity, DLCO = carbon monoxide diffusion capacity, GAP = gender-age-physiology. * = from first non-Raynaud’s symptom, ^+^ = change in FVC on next clinical follow-up visit, ° = progression defined as a decrease of FVC %predicted of ≥10% on the next follow-up visit.

| **Characteristics** | | | **Stable SSc-ILD° (n=27)** | **Progressive SSc-ILD° (n=7)** | **All (n=34)** |
| --- | --- | --- | --- | --- | --- |
| General | | |  |  |  |
|  | Female, n (%) | | 21 (77.8) | 6 (85.7) | 27 (79.4) |
|  | Age (mean±SD) | | 56.1±14.9 | 57.5±14.2 | 56.4±14.2 |
| SSc | | |  |  |  |
|  | SSc disease duration*, years (mean±SD) | | 11.0±12.3 | 6.5±5.0 | 10.1±11.2 |
|  | mRSS (mean±SD) | | 9.7±10.1 | 11.3±6.1 | 10.0±9.4 |
|  | Extent of skin disease, n (%) | |  |  |  |
|  |  | dcSSc | 14 (51.9) | 4 (57.1) | 18 (52.9) |
|  |  | lcSSc | 7 (25.9) | 3 (42.9) | 10 (29.4) |
|  |  | No skin involvement | 6 (22.2) | 0 (0.0) | 6 (17.6) |
|  | Scl-70 autoantibody positivity, n (%) | | 7 (25.9) | 4 (57.1) | 11 (32.4) |
| ILD | | |  |  |  |
|  | Lung function parameters, % predicted (mean±SD) | |  |  |  |
|  |  | FVC | 93.3±20.9 | 95.1±22.5 | 93.7±20.9 |
|  |  | DLCO | 58.5±16.7 | 60.9±13.5 | 59.0±16.0 |
|  | FVC change^+^, % (mean±SD) | | 1.2±6.6 | -18.8±8.2 | -2.9±10.7 |
|  | GAP index (mean±SD) | | 1.6±1.4 | 1.6±1.4 | 1.6±1.4 |

For SSc-ILD patients from the Cochin Hospital in Paris (n=34), the mean age was 56.4±14.2 years and, like in the University Hospital Zurich’s cohort, the majority of patients was female (n=26, 76.5%). The mean modified Rodnan skin score (mRSS) was 10.0±9.4, with 59.9% (n=18) of the patients suffering from diffuse cutaneous involvement. Patients had an average forced vital capacity (FVC% predicted) of 93.7±20.9 and a mean diffusion capacity for carbon monoxide (DLco% predicted) of 59.0±16.0. The mean disease duration from first non-Raynaud’s symptoms was 10.1±11.2 years. Clinical characteristics of patients from the French cohort are specified in Supplementary Tab. S4.

**Supplementary Table S5:** Clinical characteristics of primary myositis patients at the time of sample collection. PM = polymyositis, DM = dermatomyositis, CRP = C-reactive protein, CK = creatinine kinase. * = active disease defined as CK≥170 U/l.

| **Characteristics** | | | **Inactive disease* (n=16)** | **Active disease* (n=13)** | **All (n=29)** |
| --- | --- | --- | --- | --- | --- |
| General | | |  |  |  |
|  | Female, n (%) | | 13 (81.2) | 10 (43.8) | 23 (79.3) |
|  | Age (mean±SD) | | 59.5±10.5 | 58.7±11.0 | 59.2±10.5 |
| Myositis | | |  |  |  |
|  | Disease subtype, n (%) | |  |  |  |
|  |  | PM | 1 (6.3) | 5 (38.5) | 6 (20.7) |
|  |  | DM | 12 (75.0) | 4 (30.8) | 16 (55.2) |
|  |  | other | 3 (18.8) | 4 (30.8) | 7 (24.1) |
| Laboratory markers | | |  |  |  |
|  | CRP, mg/l (mean±SD) | | 3.0±4.3 | 2.9±4.8 | 2.9±4.4 |
|  | CK, U/l (median±IQR) | | 84.5±42.8 | 380±398.5 | 119.0±258.5 |

Patients with primary myositis (n=29) were also predominantly female (72.4%, n=21) and the mean age was 59.2±10.5. Median creatinine kinase (CK) activity at the time of blood withdrawal was 119.0±258.5 U/l, with 44.8% (n=13) patients having pathologically high CK levels. Mean serum C-reactive protein (CRP) was 2.9±4.4 mg/dl with 20.7% (n=6) of the patients presenting with levels exceeding the reference limit of 5.0 mg/dl. Clinical characteristics of myositis patients are shown in Supplementary Tab. S5.

**Supplementary Figure S1:** Pearson correlation of L-leucine (a), xanthosine (b), and branched-chain amino acids (BCAAs, c). * = p≤0.05, ** = p≤0.01, *** = p≤0.001, n.s. = p>0.05


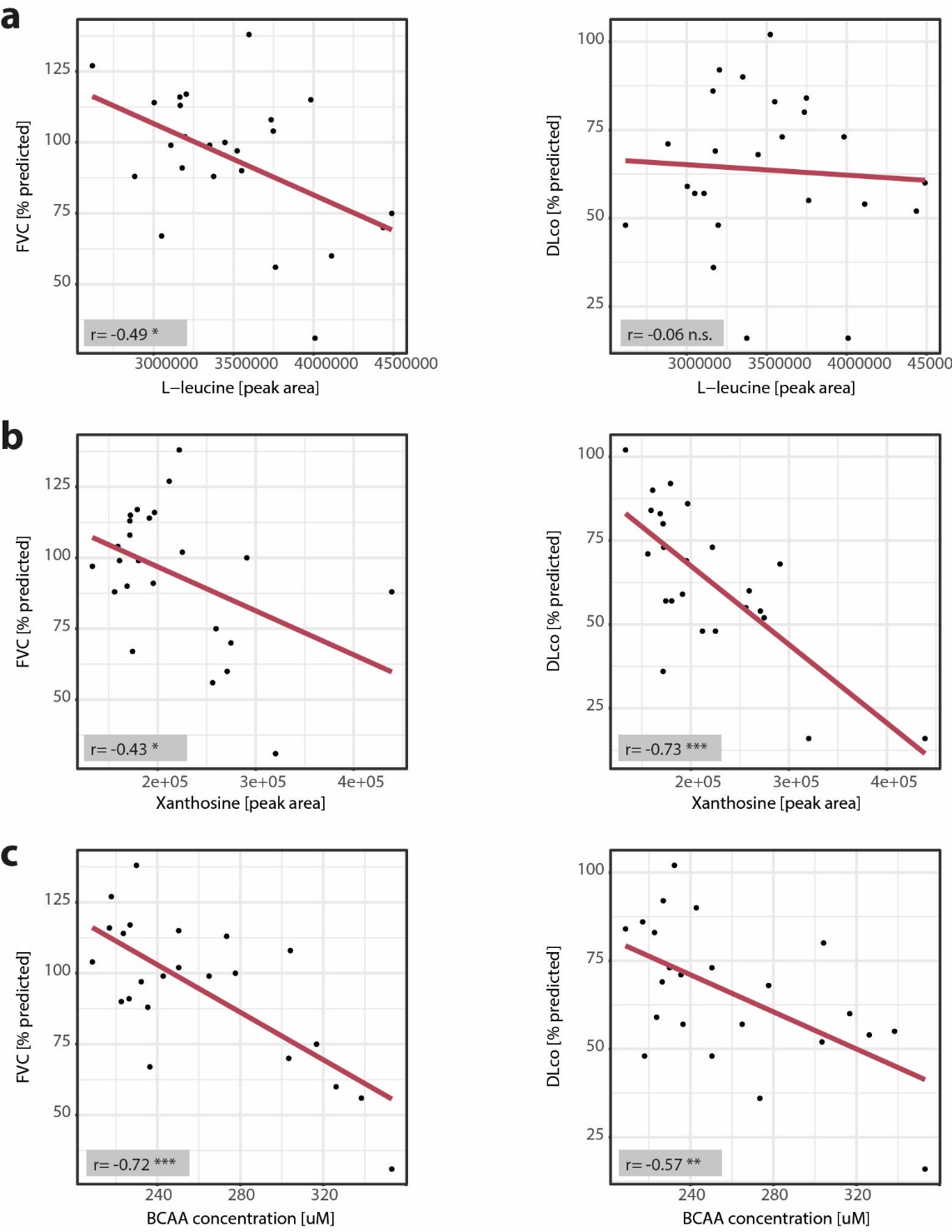


.

**Supplementary Figure S2:** Analysis of BCAA levels in the Paris cohort of SSc-ILD patients with comparison between future progressors and stable patients ((a), Student’s t-test; (b), ROC analysis) and Pearson’s correlation analysis between BCAA levels and baseline DLco (c) and GAP-index (d). * = p≤0.05, ** = p≤0.01, *** = p≤0.001, n.s. = p>0.05. N=7 (progressive) and 27 (stable).
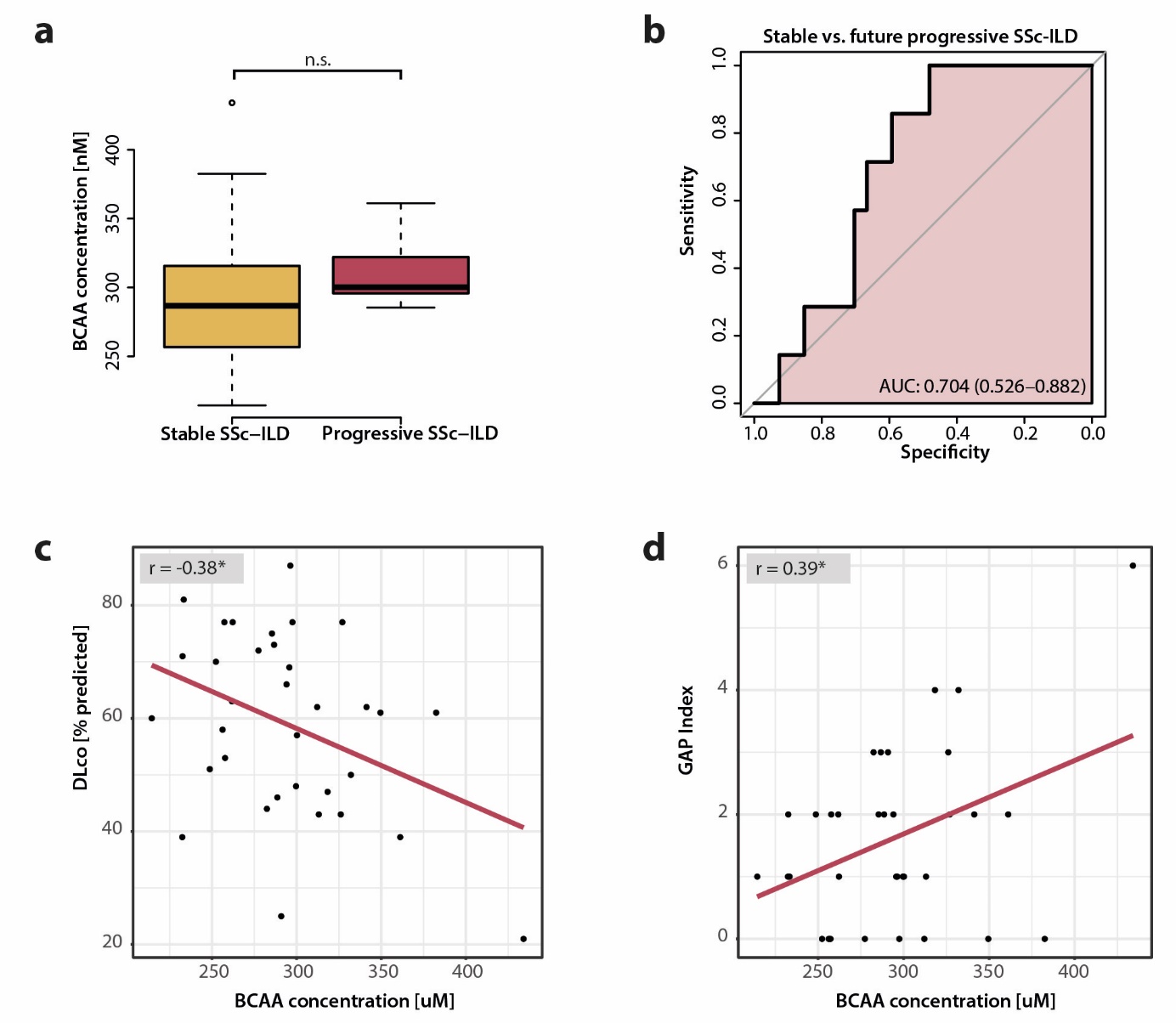


**Supplementary Figure S3:** Analysis of BCAA levels in patients with primary myositis, comparing active (n=13) and inactive (n=16) disease as defined by pathologically elevated CK levels ((a), Student’s t-test; (b), ROC analysis) and Spearman’s correlation analysis between BCAA levels and baseline CK (c) and Pearson’s correlation analysis between BCAA and CRP levels (d). * = p≤0.05, ** = p≤0.01, *** = p≤0.001, n.s.=p>0.05.
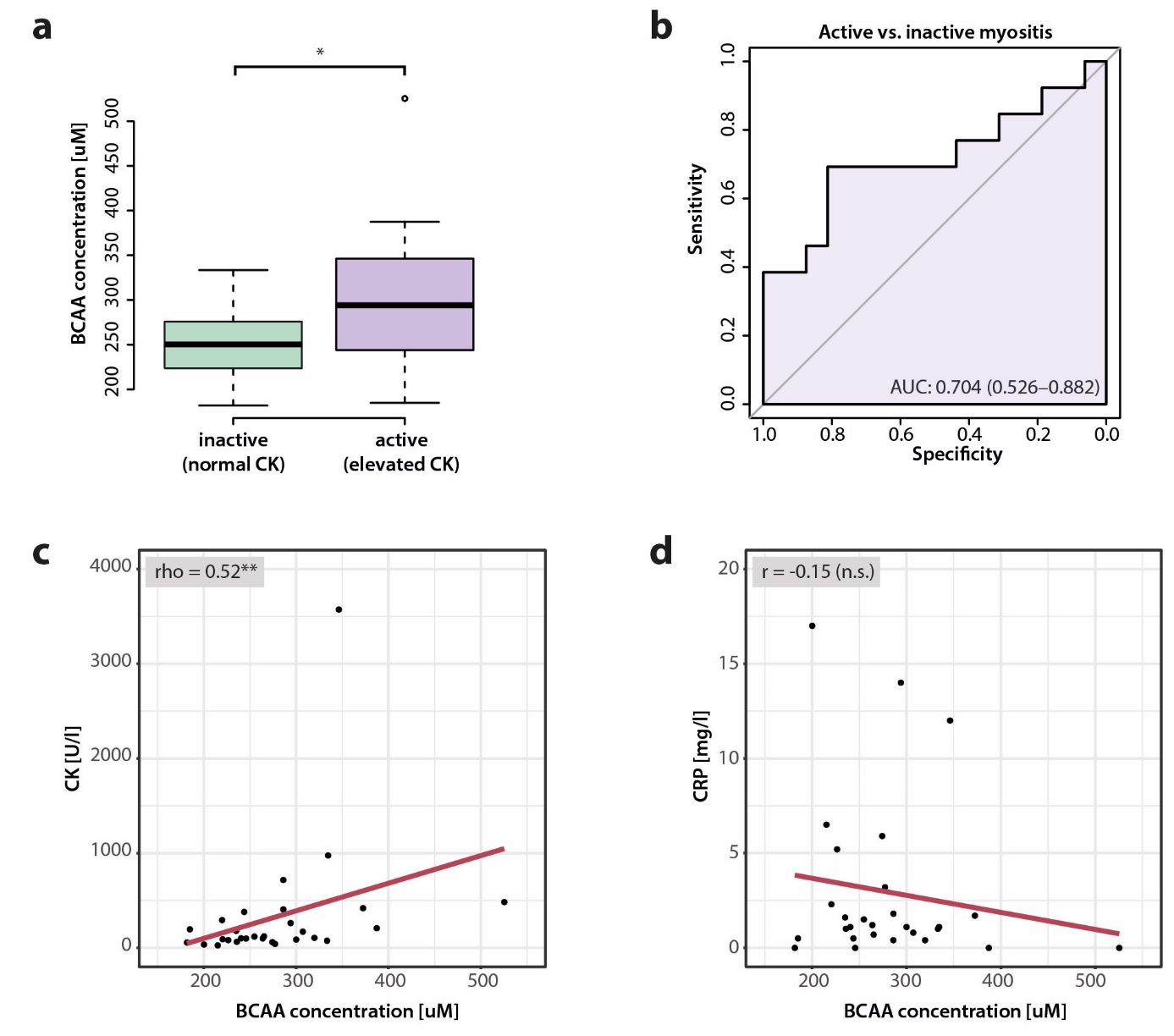


**Supplementary Methods**

**Additional patients’ cohorts for BCAA analysis**

*University Hospital Zurich - myositis patients*

Twenty-nine patients with primary myositis, but without interstitial lung disease, were included in the study. Patients were stratified into active (n=13) and inactive (n=16) disease based on blood CK levels. Active disease was defined as CK levels exceeding the upper limit of normal (≥170 U/l). Physiological reasons for elevated CK levels had been excluded.

*Cochin Hospital Paris – SSc-ILD patients*

Serum BCAA levels were additionally assessed in a cohort of SSc-ILD patients from the Cochin Hospital in Paris, who were divided into future progressors (n=7) and stable (n=27) patients based on a FVC% decline of ≥10% compared to the next follow-up visit (mean follow-up interval = 14 months (range = 5 - 20)).
